# Surgical resection and outcome of pancreatic cystic neoplasms in China: analysis of a 16-year experience from a single high-volume academic institution

**DOI:** 10.1186/1477-7819-12-228

**Published:** 2014-07-19

**Authors:** Xueli Bai, Longyun Ye, Qi Zhang, Pankaj Prasoon, Ji Wang, Tingbo Liang

**Affiliations:** 1 Department of Hepatobiliary-Pancreatic Surgery, Second Affiliated Hospital, School of Medicine, Zhejiang University, 88 Jiefang St, Hangzhou 310009, Zhejiang, China

**Keywords:** Pancreatic cystic neoplasms, Surgical resection, Malignancy, Preoperative diagnosis, Postoperative complication

## Abstract

**Background:**

To investigate the clinicopathological features of surgically resected pancreatic cystic neoplasms (PCNs) at a single institution in China.

**Methods:**

The medical charts of patients who operated in the Second Affiliated Hospital, Zhejiang University School of Medicine between 1 January 1997 and 30 June 2013, were pathologically shown to have PCNs.

**Results:**

There was a reliable increase trend not just in the overall number of patients (3 to 75) but additionally in the number of incidentally diagnosed patients across the periods (33.3% to 48.0%). In 83 of 111 cases, preoperative diagnoses matched with pathology, whereas the remaining cases (16/28) were misdiagnosed as pancreatic cancer. The proportion of malignancy in mucin producing neoplasms was 24.3% (9 out of 37). Elevated serum carbohydrate antigen (CA19-9) or carcinoembryonic antigen (CEA) was independently associated with malignancy. The overall survival rate was 96.4%.

**Conclusions:**

The proportion of PCNs within this series differs with that revealed in Western countries. Appropriate preoperative differential diagnosing of PCNs remains challenging. It is strongly recommended that patients with elevated CA19-9 or CEA levels undergo surgical resection.

## Background

The widespread use of abdominal imaging in China in the past decade has resulted in a dramatic increase in the identification of pancreatic cystic neoplasms (PCNs). This is similar to Western series such as the United States, in which cross sectional imaging has been used extensively for a longer period of time. In the Western series it has been reported that 2.6% of patients undergoing an abdominal CT scan are found to have a cystic neoplasm and another study reports 13% of patients undergoing abdominal MRI scans have cystic neoplasms [1-4]. The incidence of pancreatic cystic lesions is about 1 in 100 hospitalized patients in the United States [5].

PCN exists as a spectrum of diseases that range from completely benign to frankly malignant. Thus, careful selection for operative intervention is necessary for optimal outcomes in the management of patients with PCN. Currently, guidelines exist that guide selection for resection and are followed by most experienced centers [6]. These criteria are based on clinical features, imaging characteristics, and findings on endoscopic ultrasound/fine needle aspiration (EUS/FNA).

To date very few Chinese studies concerning surgical treatment of PCNs have been reported. It is likely that based on differences in genetic backgrounds, risk factors, and use of cross sectional imaging, significant difference exists in the incidence and indications for resection of PCN between Chinese and Western patients. Therefore the goal of this study is to report the experience of patients undergoing resection of a PCN at a single university-based high-volume pancreatic surgery center in China. In this study, we retrospectively reviewed and analyzed the data for 111 patients who underwent operations and were pathologically confirmed to have four major types of PCNs: intraductal papillary mucinous neoplasm (IPMN), mucinous cystic neoplasm (MCN), serous cystic neoplasm (SCN), and solid pseudopapillary neoplasm (SPN). To our knowledge, this is the largest series of surgically resected PCNs reported in English from China to date.

## Methods

This study was approved by the Ethics Committee of the Second Affiliated Hospital, School of Medicine, Zhejiang University and was performed according to the Declaration of Helsinki [7]. Records of patients who underwent surgical resection and were pathologically proven to have IPMN, MSC, SCN, or SPN between 1 January 1997, and 30 June 2013 at the Second Affiliated Hospital of Zhejiang University School of Medicine (China) were retrospectively reviewed and analyzed. The preoperative diagnosis, which was based on clinical manifestations, serum tumor markers, and the results of imaging including computed tomography (CT), magnetic resonance imaging (MRI) or magnetic resonance cholangiopancreatography (MRCP), were compared with the postoperative pathological diagnosis.

A serum carbohydrate antigen 199 (CA19-9) level of more than 37 U/L and a carcinoembryonic antigen (CEA) level of more than 5 ng/mL were considered to be elevated. Pancreatic fistula was diagnosed according to the International Study Group on Pancreatic Fistula criteria [8].

In the final pathologic reports, cystic neoplasms were classified according to the World Health Organization classification of the exocrine and endocrine neoplasms of the pancreas [9]. IPMN and MCN were graded based on degree of dysplasia: low-grade, intermediate grade, high-grade (carcinoma *in situ* (CIS)), and invasive. Serous cystadenocarcinoma was defined by the presence of metastases [10].

Information about recurrence and survival time was obtained via follow-up phone calls and clinical interviews. Statistical analysis was carried out using SPSS version 19.0 (SPSS, Chicago, Illinois, United States). Continuous variables were expressed as medians and ranges and compared using the Mann-Whitney test. Categorical variables were compared using a chi-squared test (or Fisher’s exact test). Univariate analyses were conducted using chi-square or Mann-Whitney *U* tests, as appropriate, and multivariate analysis was performed using forward step-wise logistic regression analysis. Survival analysis was performed using the Kaplan-Meier method, with differences determined by the log-rank test. All tests were two-sided and *P* <0.05 was considered to indicate statistical significance.

## Results

### Demographic data and clinical features

**Figure 1:**
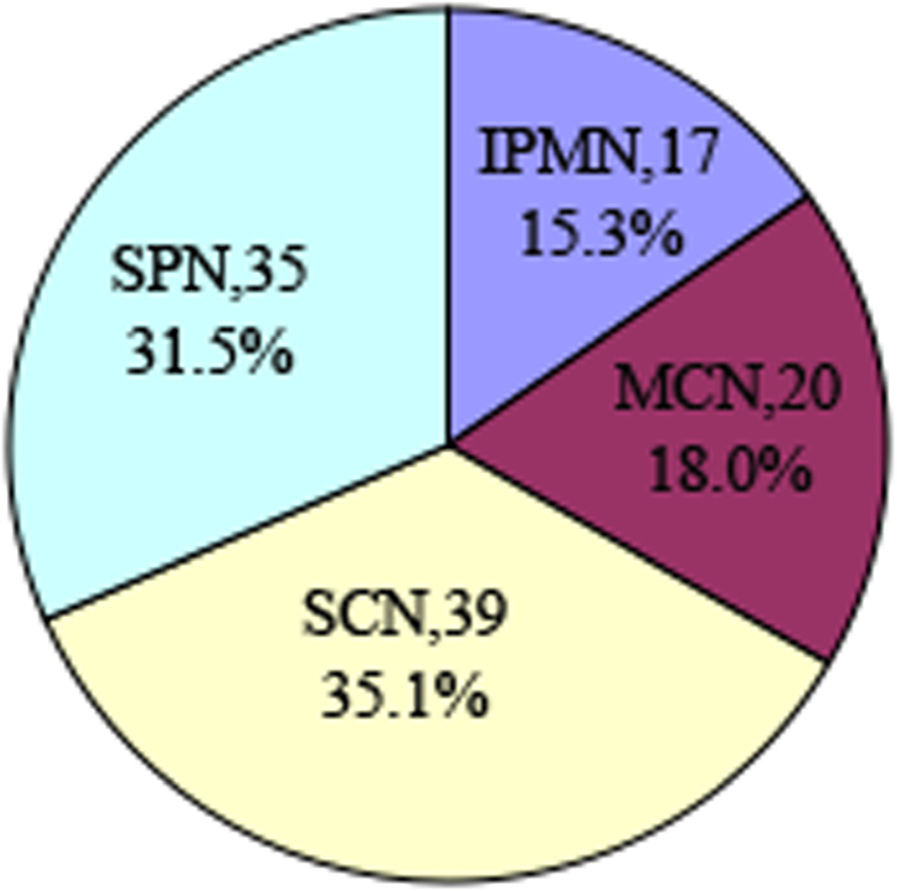
Distribution of PCNs in 111 patients.

A total of 111 patients were enrolled in this study, including 17 (15.3%) with IPMNs, 20 (18.0%) with MCNs, 39 (35.1%) with SCNs, and 35 (31.5%) with SPNs (Figure 1). All patients were of Chinese ethnicity. The age of the patients varied from 13 to 81 years, with the median age of 46 years, and 75.7% were female (Table 1). Patients undergoing resection of SPN (median age: 30 years; range: 13 to 59 years) were significantly younger than those in the IPMN group (median age: 71 years; range: 44 to 81 years; *P* <0.001), MCN group (median age: 48 years; range: 24 to 75 years; *P* <0.001) and SCN group (median age: 53 years; range: 24 to 78 years; *P* <0.001). Meanwhile, the patients with IPMN (median age: 71 years; range: 44 to 81 years) were older than the other patients (*P* <0.001). Females were more common among SPN (91.4%, 32 out of 35), MCN (100%, 20 out of 20) and SCN (71.8%, 28 out of 39), while IPMN noted a slight male dominance (76.5%, 13 out of 17; *P* <0.05). Sixty-two (55.9%) patients presented symptoms at admission. Of these, the chief complaint was abdominal pain and distention in 50 patients (45.0%) and weight loss in 9 (8.1%) patients; jaundice, nausea, and tumor on palpation were found in 19 cases (17.1%). There was no significant difference among the various PCNs. Forty-nine patients (44.1%) were asymptomatic, and their cysts had been discovered incidentally either in a routine health examination or on imaging performed for another complaint. The majority of MCNs (90.0%, 18 out of 20) occurred in the body and tail of the pancreas, whereas SCNs and SPNs tended to be evenly distributed. IPMNs (70.6%, 12 out of 17) were far more likely to be located in the head. MCNs were significantly larger compared with other cystic neoplasms, with a median length of 6.0 cm (range: 2.8 to 18.0 cm; *P* <0.05) and width of 5.0 cm (range: 2.0 to 10.0 cm; *P* <0.05).

**Table 1:** Clinical data for the 111 pancreatic cystic neoplasms

	**IPMN (n = 17)**	**MCN (n = 20)**	**SCN (n = 39)**	**SPN (n = 35)**
Age, years, median (range)	71 (44-81)	48 (24-75)	53 (24-78)	30 (13-59)
Gender, n (%)				
Male	13 (76.5%)	0 (0%)	11 (28.2%)	3 (8.5%)
Female	4 (23.5%)	20 (100%)	28 (71.8%)	32 (91.4%)
Symptoms, n (%)	13 (76.5%)	12 (60%)	21 (53.8%)	16 (45.7%)
Abdominal pain and distension	9	8	18	15
Weight loss	3	2	3	1
Jaundice	2	0	1	0
Nausea	1	1	2	5
Tumor palpation	1	4	1	1
No symptoms, n (%)	4 (23.5%)	8 (40%)	18 (46.2%)	19 (54.3%)
Tumor location, n (%)				
Head and/or uncinate process	12 (70.6%)	1 (5.0%)	16 (41.0%)	14 (40.0%)
Neck	2 (11.8%)	1 (5.0%)	6 (15.4%)	3 (8.6%)
Body and/or tail	3 (17.6%)	18 (90.0%)	17 (43.6%)	18 (51.4%)
Tumor size, cm, median (range)				
Length	3.4 (1.8-15.0)	6.0 (2.8-18.0)	4.5 (1.2-15.0)	4.0 (1.2-15.0)
Width	2.6 (1.0-10.0)	5.0 (2.0-10.0)	3.0 (1.2-10.0)	3.5 (1.2-8.0)

### Trends in the incidence and types of PCNs

In our study, patient characteristics were analyzed and compared among four periods (Table 2) from 1997 to 2013: 1997 to 2000, 2001 to 2004, 2005 to 2008, and 2009 to 2013. We found a step-wise increase not only in the total number of patients but also in the number of incidentally diagnosed patients. The number of patients who were incidentally diagnosed in the last four years (2009 to 2013) was 36, which is a three-fold increase compared to the 2005 to 2008 period. In the same period (2009 to 2013), almost one-half of the resected PCNs were found incidentally, although it did not reach a significant difference. The median time lapse from the presentation of symptoms and diagnosis of the cystic lesions of the pancreas was one month (range: 0.02 to 144), no significant difference was found between these time periods.

**Table 2:** Trends in surgically resected pancreatic cystic neoplasms

	**1997-2000**	**2001-2004**	**2005-2008**	**2009-2013**
N	3 (2.7%)	6 (5.4%)	27 (24.3%)	75 (67.6%)
Symptoms, n (%)				
Yes	2 (66.7%)	4 (66.7%)	17 (63.0%)	39 (52.0%)
No	1 (33.3%)	2 (33.3%)	10 (37.0%)	36 (48.0%)
Time interval, months, median (range)*	0.3 (0.2-12.0)	1 (0.2-48.0)	1 (0.1-120.0)	1 (0.02-144.0)
Pancreatic cystic neoplasms, n (%)				
IPMN (intraductal papillary mucinous neoplasm)	0	0	5 (18.5%)	12 (16.0%)
MCN (mucinous cystic neoplasm)	2 (66.7%)	1 (16.7%)	3 (11.1%)	14 (18.7%)
SCN (serous cystic neoplasm)	1 (33.3%)	1 (16.7%)	15 (55.6%)	22 (29.3%)
SPN (solid pseudopapillary neoplasm)	0	4 (66.7%)	4 (14.8%)	27 (36.0%)

*The time lapse from the presentation of symptoms and diagnosis of the cystic lesions of the pancreas.

Additionally, the proportion of the pathological types of PCNs varied with time. During 2009 to 2013, SPN and SCN each comprised 36.0% and 29.3% of PCNs; however, in 2005 to 2008 SCN was the main pathological type, accounting for 55.6% of PCNs (*P* <0.05), and SPNs only accounted for 14.8% of PCNs (*P* <0.05). IPMNs also showed an increasing trend over time: only 5 patients were diagnosed before 2008, while it was confirmed in 12 patients in 2009 to 2013, although it did not reach a statistically significant difference.

### Preoperative diagnostic accuracy

In the preoperative work-up, an abdominal CT scan was performed in 104 of 111 patients (93.7%), and an MRI or MRCP was used in 27 (24.3%) patients. In addition, EUS/FNA and endoscopic retrograde cholangiopancreatography (ERCP) were performed in 2 (1.8%) and 1 (0.9%) patients, respectively.

Preoperative diagnoses of 83 patients were approved by pathology, while the remaining 21 (18.9%, 21/111) were misdiagnosed as pancreatic cancer (in 16 patients), pseudocyst (in 4 patients) and neuroendocrine tumor (in 1 patient). Seven (6.3%, 7/111) patients were indistinguishable from other PCNs (Table 3).

**Table 3:** Preoperative diagnosis of pancreatic cystic neoplasms

	**IPMN (n = 17)**	**MCN (n = 20)**	**SCN (n = 39)**	**SPN (n = 35)**
Preoperative diagnosis, n, (%)	11 (64.7%)	17 (85%)	28 (71.8%)	27 (77.1%)
Misdiagnosis, n, (%)	6 (35.3%)	3 (15%)	11 (28.2%)	8 (22.9%)
Carcinoma	5 (83.3%)		4 (36.5%)	7 (87.5%)
Neuroendocrine tumor			1 (9%)	
Pseudocyst	1 (16.7%)	3 (100%)		
Others PCNs			6 (54.5%)	1 (12.5%)

### Surgical intervention and operative complications

A surgical intervention was performed because of the presence of symptoms in 62 patients. In the other 49 (44.1%) asymptomatic patients, resection was driven by the suspected malignancy or uncertain diagnosis with tumor size of larger than 3 cm.

In 39 SCNs, 21 patents (53.8%) were operated on because of clinical symptoms and 11 were operated on because of large tumors (more than 4 cm) [11]. Four patients were misdiagnosed as pancreatic cancer, and 3 patients’ tumors were indistinguishable from MCNs before the operation.

In total, 50 (45.0%) distal pancreatectomies (of which 14 procedures were done with preservation of the spleen and 4 were performed laparoscopically), 26 (23.4%) pancreaticoduodenectomies, 17 (15.3%) middle pancreatectomies, 15 (13.5%) enucleations, 1 (0.9%) pylorus-preserving pancreaticoduodenectomy, 1 (0.9%) total pancreatectomy, and 1 (0.9%) laparotomy with a diagnostic biopsy were performed (Table 4).

**Table 4:** Surgical interventions and morbidity

	**IPMN (n = 17)**	**MCN (n = 20)**	**SCN (n = 39)**	**SPN (n = 35)**
Types of resection, n (%)				
Distal pancreatectomy, splenectomy	2 (11.8%)	11 (55%)	11 (28.2%)	12 (34.3%)
Spleen-preserving distal pancreatectomy	2 (11.8%)	6 (30%)	3 (7.7%)	3 (8.6%)
Pancreaticoduodenectomy	11 (64.7%)		6 (15.4%)	9 (25.7%)
Pylorus-preserving pancreaticoduodenectomy			1 (2.6%)	
Middle pancreatectomy			12 (30.8%)	5 (14.3%)
Tumor enucleation	1 (5.9%)	2 (10%)	6 (15.4%)	6 (17.1%)
Laparotomy with biopsy		1 (5%)		
Total pancreatectomy	1 (5.9%)			
Postoperative complications, n (%)	8 (47.1%)	6 (30%)	15 (38.5%)	21 (60%)
No complications, n (%)	9 (52.9%)	14 (70%)	24 (61.5%)	14 (40%)
Re-operation (n)	0	0	2 (5.1%)	2 (5.7%)
Hospital stay after surgery, days, median (range)	19.0 (12.0-74.0)	13.0 (8.0-26.0)	20.0 (7.0-123.0)	16.0 (7.0-99.0)

Postsurgical complications occurred in 50 patients (45.0%), among whom the most common complication was pancreatic fistula (29.7%, 33 out of 111). The incidence of pancreatic fistula was higher in the SPN group (42.9%, 15 out of 35), but was not significantly different between the other groups (*P* >0.05). Other complications included intra-abdominal infection (14.4%, 16 out of 111), pleural effusion (11.7%, 13 out of 111), delayed gastric emptying (8.1%, 9 out of 111), pulmonary infection (6.3%, 7 out of 111), bile leakage (3.6%, 4 out of 111) and wound infection (2.7%, 3 out of 111). Furthermore, reoperation was necessary in 4 patients (3.6%, 4 out of 111); 2 due to hemorrhages, 1 due to bile leakage, and 1 due to pancreatic fistula. The mortality rate in this series was 0.9% (1 out of 111): one patient in the IPMN group who underwent distal pancreatectomy died on the 22nd postoperative day because of pneumonia and septic shock. The median hospital stay after surgery was 17.0 days (range: 7 to 123 days) in 110 patients. In the MCN group, this was 13.0 days (range: 8.0 to 26.0 days), which was significantly shorter than that in the IPMN group (median: 19.0 days; range: 12.0 to 74.0 days; *P* <0.05), SCN group (median: 20.0 days; range: 7.0 to 123.0 days; *P* <0.05) and SPN group (median: 16.0 days; range: 7.0 to 99.0 days; *P* <0.05).

### Predictive factors of malignancy in mucin-producing neoplasms

In mucin-producing neoplasms (IPMNs and MCNs), malignancy was found 9 patients, 6 in IPMNs, and 3 in MCNs. Univariate analysis revealed gender and elevated CA19-9 or CEA level to be significant predictive factors of malignance (Table 5). Multivariate analysis revealed that elevated CA19-9 or CEA levels were independent predictive factors of malignancy (*P* <0.05) (Table 6).

**Table 5:** Univariate analysis of predictive factors of malignant pancreatic cystic neoplasms

	**Malignant (n = 9)**	**Benign (n = 28)**	***P *** **value**
Age, years, median (range)	64 (50-77)	49 (24-81)	0.051
Gender (n)			0.036
Male	6	6	
Female	3	22	
Symptoms			0.446
Yes	7	17	
No	2	11	
Tumor location (n)			0.573
Head and/or uncinate process	4	8	
Neck	1	2	
Body and/or tail	4	18	
PCN classification			0.251
IPMN (intraductal papillary mucinous neoplasm)	6	11	
MCN (mucinous cystic neoplasm)	3	17	
Elevated serum carbohydrate antigen (CA19-9) or carcinoembryonic antigen (CEA)			0.014
Yes	7	5	
No	2	15	
Tumor size (cm)			0.403
>3 cm	8	20	
≤3 cm	1	8	

**Table 6:** Multivariate analysis of the predictive factors of malignant pancreatic cystic neoplasms

**Variable**	***p*-value**	**Odds ratio (OR)**	**95% CI**
Gender	0.998	N/A	N/A
Elevated serum carbohydrate antigen (CA19-9) or carcinoembryonic antigen (CEA)	0.011	0.089	0.014-0.576

N/A not available.

### Follow-up data

**Figure 2:**
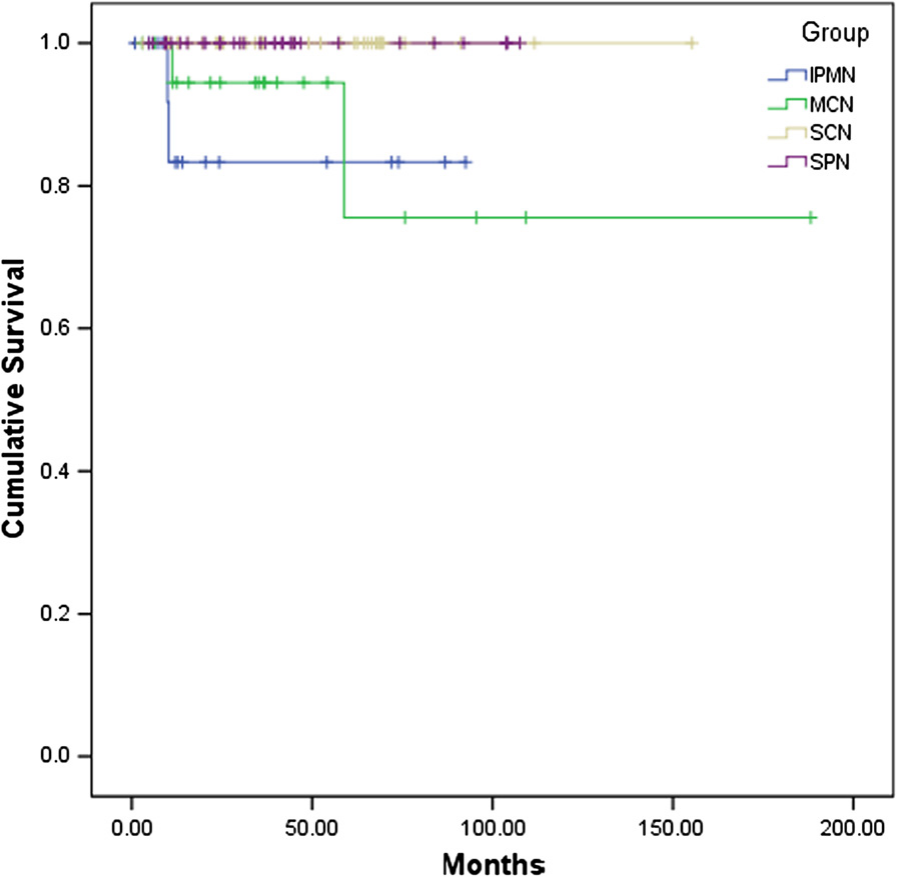
Kaplan-Meier overall survival curves of patients who underwent surgical operations for pancreatic cystic neoplasms.

Survival data were collected from all 110 patients, who were followed-up for a median time of 35.67 months (0.9 to 188.23 months). The overall survival rate was 96.4%. Survival analysis showed that the survival rate of SPNs (100%) and SCNs (100%) was significantly better than that of IPMNs (87.5%) and MCNs (90.0%) (Figure 2).

## Discussion

In this study we have obtained comprehensive data on the trends in PCNs in one single institution in China over the last 16 years. We found an increase in the number of surgically managed PCN cases in the last four years. This is similar to the data for other countries. For example, PCNs were found to represent at least 25% of resections at the major pancreatic referral centers in the United States in 2003 [12].

A detailed review of previous studies has shown that there is a difference in the relative proportions of different pathologic diagnoses. Coelho *et al*. [13] reported in 2010 that only 4 (15%) patients were diagnosed IPMN, and in a series of 599 consecutive patients, Bassi *et al*. [14] found an IPMN rate of 17%. In our study, IPMN was only diagnosed in 15.3% of the patients. In contrast to this, a recent study at Massachusetts General Hospital demonstrated that IPMN was the most common type of PCN resected and accounted for nearly one-half of the resected cystic neoplasms in the last five years [15]. Main duct and/or mixed-duct and branch-duct IPMN were also reported to comprise 48.6% of PCNs in the Department of Surgery, University of Verona, Italy, in 2012 [16]. This disagreement between the results may be explained by the following: (1) Due to economic issues, abdominal cross-sectional imaging examination with CT or MRI is not routinely performed in China so far. While most IPMNs are asymptomatic, this may lead to a low detection rate of IPMN. (2) The recognition of IPMN by a pathologist in China is probably not as good as in Western countries. Under these circumstances, IPMN may be misdiagnosed as MCN or pancreatic adenocarcinoma. According to a recent publication on 2564 resected periampullary adenocarcinomas at Johns Hopkins’s hospital, the frequency of pancreatic cancer arising from IPMN was 8% during the 2000s [17]. We cannot exclude the possibility that pancreatic cancers arising from IPMNs have been pathologically diagnosed as cancer while neglecting theirIPMN origin, which may also attribute to the low frequency of IPMN in our series. (3) To date, there are very few publications on PCN from China and the real proportion of each type in China is still unknown. Whether there is a racial difference-related proportion is need to be further investigated. The accuracy of preoperative diagnosis of IPMN was 64.7% at our hospital during our 16-year experience, whereas the rate of correct diagnosis for IPMN was 80% in the series in Salvia *et al*. [16].

IPMN was first differentiated from mucin-producing cystic neoplasms by Ohashi and Murakami in 1990 [18]; before this, the detection rate of IPMNs was only 3% [19]. It has been established that MCNs do not generally communicate with the pancreatic ducts, a macroscopic feature used in many series as a factor to determine MCN from IPMN [19]. The sole criterion for the diagnosis of MCN is the presence of well-demarcated cysts lined by a mucin-producing columnar epithelium overlying an ovarian-type stroma. Therefore, some authors re-evaluated pathology slides from previous reports. Niedergethmann *et al*. [20] reported in 2008 that the incidence of incorrect diagnosis was greater in the earlier time period (1996 to 2001) of their study, so they eventually reclassified 54 neoplasms as IPMN among 207 cases, which was 125% more than the originally diagnosed number. In another study, Tollefson *et al*. [21] also found that 21 cases of IPMN were misdiagnosed among 84 cases between 1960 and 1980 at the Mayo Clinic (Rochester, Minnesota).

The incidence of SPN reported here is also quite different from that in other reports. SPN is a rare tumor; it forms 1 to 3% of all pancreatic tumors and 5% of all cystic tumors of the pancreas according to previous results [22]. This results concur with those of Valsangkar *et al*.’s analysis [14] in which only 29 (3.4%) of the patients were diagnosed with an SPN. The data from another retrospective study of 476 patients who underwent pancreatic resection also documented a lower incidence (8.0%) in 2012 [16]. Similarly, Bassi *et al*. [14] reported that the incidence of SPN was 6.5%. On the contrary, a higher prevalence of SPN (31.5%, 35 out of 111) was found in our series, with 77.1% of the patients diagnosed in 2009 to 2013. There also has been a higher incidence in the number of SPNs reported in another case series in China (17.3%) in 2005 [23]. Furthermore, a multi-institutional study in Korea conducted over 13 years that included 1064 cases of pathologically confirmed PCNs, showed that the incidence of SPN was 18.3% in 2008 [24]. Several large single-institution series on SPN have been reported about in other Asian countries such as Singapore [25], Taiwan [26], India [27], and Japan [28]. This apparent increase in the incidence of SPNs in our study may represent a true increase in incidence resulting from unknown environmental or genetic factors in Asia. On the other hand, there may be possible selection bias; since we included cases of surgically resection only, the increase in the SPN rate may be overestimated as most other PCNs are managed by conservative treatment.

Surgical intervention generally depends on the location and size of the tumor [13]. Tumors of the head and uncinate process should be treated with pancreatoduodenectomy. Small lesions can also be managed with middle pancreatectomy or tumor enucleation, sparing the pancreatic tissue. Distal pancreatectomy, with or without spleen preservation, should be performed for PCNs identified in the body and tail of the pancreas [29].

In our study, the mortality rate was 0.9%; that is, only one patient died in the perioperative period as a result of pulmonary infection. The overall complication rate was 45.0%. These results are similar to those reported in other literature [15,30]. The most common complication was pancreatic fistula; its incidence was highest in the patients who underwent middle pancreatectomy, but there was no significant difference between these surgical treatments. Therefore, middle pancreatectomy is recommended in cases where major pancreatic resection, such as pancreaticoduodenectomy, is not required

PCNs encompass a wide spectrum of benign, borderline, and malignant diseases; the preoperative identification of predictors for malignancy is very critical for subsequent management. Following the primary assessment of the clinical presentation, tumor markers and imaging examinations are recommended for preoperative evaluation. PCNs with elevated serum CEA or CA19-9 should be treated with surgical resection because of the high risk of harboring a potentially tumor or malignant lesion [29]. Our statistical analyses confirmed that an elevated CEA and/or CA19-9 is an independent predictive factor for malignant pancreatic cysts (*P* = 0.011). In radiological studies, mural nodules, symptoms associated with the cyst, thick septa, peripheral calcification, and concomitant dilation of the pancreatic duct are features associated with malignancy [1]. CT and MRI scans are both capable of predicting the presence of malignancy in pancreatic cysts more accurately (73 to 79%) than other imaging methods in use [31]. In China, EUS is much more commonly used to perform FNA in order to provide fluid for cytological analysis and tumor markers or amylase testing, which plays an important role in diagnosis of PCNs. Although there was a marked increase in the use of EUS/FNA, EUS alone has been shown to be inadequate for differentiating neoplastic cysts from non-neoplastic cysts because of the considerable overlap between mucinous and non-mucinous cysts [16,32].

From the follow-up data, the prognosis of patients with resected PCNs seems to be favorable. None of our SPN patients died during the study period, which is consistent with the relatively indolent biological properties of this tumor. For mucin-producing tumors, a significantly lower survival rate was found in MCNs (90.0%) and IPMNs (87.5%), consistent with the reports of others [4,15].

## Conclusions

With the widespread use of high-resolution abdominal imaging, the detection rate of PCNs has increased. However, there are still obstacles in the differential diagnosis of various types of PCNs. In recent years, the mortality rate and major complications have decreased with improvements in pancreatic surgery techniques and perioperative management. Favorable long-term survival after resection of PCNs compared to adenocarcinoma has also been obtained. Therefore, aggressive surgical resection is advocated for PCN patients, especially in the case of those with symptoms, elevated CA19-9 or CEA levels, and malignancy features.

## Consent

Written informed consent was obtained from the patient for the publication of this report and any accompanying images.

## Abbreviations

CA19-9: Carbohydrate antigen 199; CEA: Carcinoembryonic antigen; IPMN: Intraductal papillary mucinous neoplasm; MCN: Mucinous cystic neoplasm; PCN: Pancreatic cystic neoplasms; SCN: Serous cystic neoplasm; SPN: Solid pseudopapillary neoplasm; CT: computed tomography; MRI: magnetic resonance imaging; MRCP: magnetic resonance cholangiopancreatography; EUS/FNA: endoscopic ultrasound/fine needle aspiration; ERCP: endoscopic retrograde cholangiopancreatography.

## Competing interests

The authors declare that they have no competing interests.

## Authors’ contributions

TBL designed the study and critically revised the manuscript. XLB and LYY collected and analyzed the clinicopathological data. XLB, LYY and QZ drafted the manuscript. PP and JW edited the data. All authors have read and approved the final version of the manuscript.
